# Dynamic Clinical Data Mining: Search Engine-Based Decision Support

**DOI:** 10.2196/medinform.3110

**Published:** 2014-06-23

**Authors:** Leo Anthony Celi, Andrew J Zimolzak, David J Stone

**Affiliations:** ^1^Harvard-MIT Division of Health Science and TechnologyInstitute for Medical Engineering and ScienceMassachusetts Institute of TechnologyCambridge, MAUnited States; ^2^Beth Israel Deaconess Medical CenterBoston, MAUnited States; ^3^Children's Hospital Informatics ProgramDepartment of PediatricsHarvard Medical SchoolBoston, MAUnited States; ^4^University of Virginia School of MedicineDepartments of Anesthesiology and NeurosurgeryCharlottesville, VAUnited States

**Keywords:** decision support, clinical informatics, big data

## Abstract

The research world is undergoing a transformation into one in which data, on massive levels, is freely shared. In the clinical world, the capture of data on a consistent basis has only recently begun. We propose an operational vision for a digitally based care system that incorporates data-based clinical decision making. The system would aggregate individual patient electronic medical data in the course of care; query a universal, de-identified clinical database using modified search engine technology in real time; identify prior cases of sufficient similarity as to be instructive to the case at hand; and populate the individual patient's electronic medical record with pertinent decision support material such as suggested interventions and prognosis, based on prior outcomes. Every individual's course, including subsequent outcomes, would then further populate the population database to create a feedback loop to benefit the care of future patients.

## Introduction

With the near universal implementation of electronic medical records (EMRs) in conjunction with enhanced data storage options, the time nears for real time data utilization in the clinical care process [1]. The subject of the increasing importance of data for health care is much talked and written about, but there is much less discussion regarding how data might specifically be used to drive and improve the individual clinician-patient interactions that accrue to formulate the process of health care. In other words, how could complete clinical decision support be implemented across the entire health care system? Big data is an increasing presence in health care, but data of all sizes are still underutilized. In those instances when they are used at all, they are used mainly in a retrospective analytic manner to analyze outcomes, processes, and costs. Currently, they do not dynamically drive clinical decision making in real time.

We have written on the need for the better use of intensive care unit data, noting that the development of data-based clinical decision support (CDS) tools would be one of the benefits of more comprehensive data capture [2]. Currently, the medical digital world comprises systems that are technically networked, but with data that are not systematically gathered, captured, or analyzed [3]. There are several studies that have demonstrated the potential applications and potential of capturing and analyzing clinical data [4,5]. In a more general response to this challenge, we describe a solution that combines the utilization of three fundamental components in real time: (1) big data, (2) search engines, and (3) EMRs. In particular, search engines are brilliant tools that we all utilize many times each day; however, they have not been systematically employed for the purpose of CDS, and they represent an overlooked resource.

## Dynamic Clinical Data Mining

**Figure 1:**
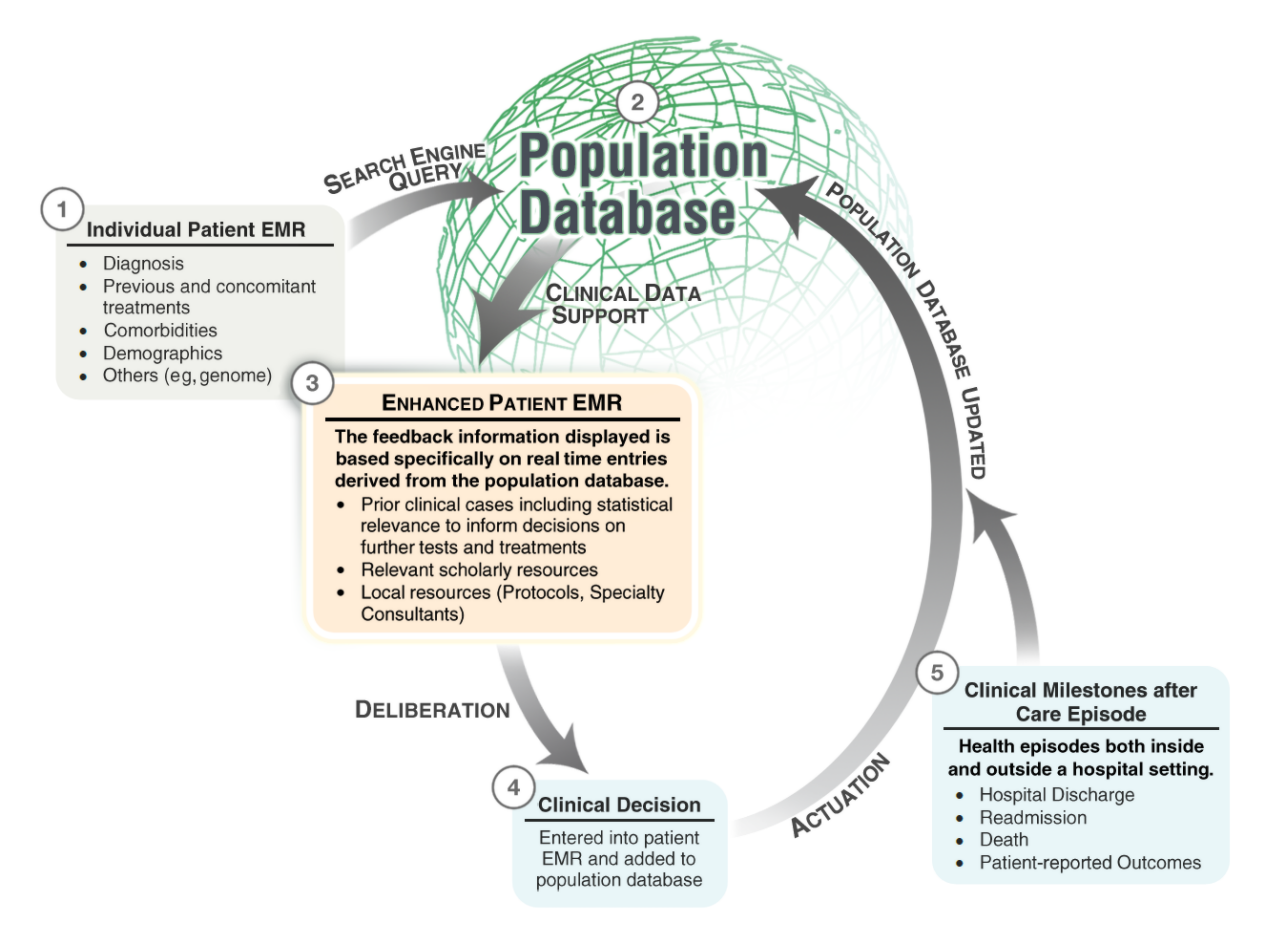
Dynamic clinical data mining. Figure courtesy of Kai-ou Tang and Edward Moseley. EMR=electronic medical record.

The struggle to implement EMRs is finally coming to a mainly successful end in North America. However, the current generation of EMRs serves to digitalize information, but not to leverage it. The next step in the clinical digitization process should be the creation of a medical Internet equivalent that incorporates the rapid, powerful data search engine features that all current Web users employ. We refer to the real time incorporation of external data into the workflow as dynamic clinical data mining (DCDM) (Figure 1 shows this mining). This process will drive the design of the next generation of EMRs, and it will subsequently support the next required stage of the digital transformation process by turning medical practice into a data driven, logical, and optimized system. This care support system will provide users with the timely information that they require to make the increasingly complex decisions of medical practice.

We propose a system in which the knowledge gained from the care of individuals systematically contributes to the care of populations. The loop is closed when the richer data available in the population datasets is subsequently used in the care of individuals. DCDM would leverage the automatic crowd sourcing available in the form of population outcome analysis to formulate individualized diagnostic and therapeutic recommendations in real time. In other words, our viewpoint aligns with the Committee on a Framework for Developing a New Taxonomy of Disease, who advocate that “researchers and health care providers have access to very large sets of health and disease-related data linked to individual patients” in order to facilitate precision medicine [6]. To the Committee’s position, we would add our own that researchers and clinicians are already experienced with Internet search engines, so they would be comfortable with the identification of pertinent clinical information by accessing these large sets of data through a search engine metaphor. Currently, most clinical guidelines are generated by expert opinion based on experience and research findings such as randomized controlled trials [7]; DCDM would formulate the functional equivalent of personalized clinical guidelines.

While leading a team in the intensive care unit (ICU), one of us (LAC) experienced a difficult decision involving the resumption of anticoagulation in a patient with two mechanical heart valves. The patient was recovering from endocarditis complicated by brain abscesses. The team consulted local experts as well as the literature to guide them in weighing the risks and benefits of reinitiating anticoagulation, given the patient’s age, comorbidities, the specific bacteria involved, the number of mechanical valves, the extent and current status of the infection, etc. The information resources that were accessed provided only general recommendations that were obviously not tailored to the patient’s demographics and comorbidities, nor to the specifics of the clinical context. The majority of these recommendations were based on expert opinions or small clinical trials, and not on “gold-standard”, multi-center randomized controlled studies. The decision was made to restart anticoagulation cautiously, given the patient’s clinical stability, the absence of bleeding complications during the acute phase, and the lack of any planned surgical intervention. In fact, preparations were underway for discharge to a skilled nursing facility. Unfortunately, four days after reinitiation of anticoagulation, the patient suffered from a massive hemorrhage of one of the brain abscesses, prompting emergent hemicraniectomy. A DCDM system could have provided predictions of the harms and benefits of anticoagulation for such a complicated patient, and it would provide the previous outcomes associated with each treatment option to review in real time [8].

Uncertainties are not limited to complex scenarios, but occur with alarming frequency in all medical settings. For example, on a daily basis in the ICU, emergency department, or the operating room, clinicians target a desired blood pressure according to population-based guidelines. When hypotension ensues, the timing, mode, and extent of intervention to maintain that goal remain art rather than science. Given that interventions to raise blood pressure such as vasopressor therapy or fluids are associated with risk of harm if given even slightly in excess, it is crucial that the targeted blood pressure be personalized as much as possible. DCDM would add the knowledge gained from prior care of populations to the current local data specifics in order to formulate an approach that is optimal in terms of both the short-term goal as well as the long-term outcomes. For instance, DCDM could assist an ICU physician in choosing an intervention and its dose to treat shock, such that the intervention has the optimal effect on the short-term blood pressure profile and long-term mortality, length of stay, and/or eventual quality of life.

Other studies have explored similar themes. Certainly the application of logic and probability to medicine has been discussed for decades [9]. More recently, and more to the point of our discussion, a variety of commentators have called for a nationwide learning health system [10,11]. In 2011, Frankovich et al reported the case of a girl with lupus and potential thrombotic risk factors. To determine whether anticoagulation was appropriate, they used text searching to retrieve records of similar patients from their hospital’s EMR, followed by a focused manual review. They found that pediatric patients with lupus and these potential risk factors indeed had a higher risk of thrombosis than those without the risk factors, and they elected to start anticoagulation as a result [12]. The Query Health initiative, from the Office of the National Coordinator for Health Information Technology, intends to facilitate distributed queries, which can aggregate results from multiple organizations’ patient populations while preserving data security [13]. Similarly, the goal of the Strategic Health Information Technology Advanced Research Project on secondary use (SHARPn) is to standardize structured and unstructured EMR data to promote its reuse [14]. The open source Clinical Text Analysis and Knowledge Extraction System and the SHARPn program use the Unstructured Information Management Architecture, the same architecture that allowed the Watson system from International Business Machines to compete on the Jeopardy! television quiz show [15,16]. In general, search engines for unstructured text are seen as the first step, and implementation of “content analytics” is the next step to extract information, allow exploration, and to improve search [17].

CDS provides caregivers with information to improve the quality of their decision making, yet caregivers still do not have available a dynamic, comparative analysis between the current patient and all available data generated during clinical care. This analysis is individually tailored because it uses EMR data entered on one specific patient, yet it remains population-based because the analysis makes a comparison to population data, to identify similar clinical situations from the past, and to mine them for interventions and subsequent outcomes (as illustrated in point 5 in Figure 1). Thus, the clinician does not have to make a decision in isolation from what has been tried, observed, and documented by many colleagues in many other similar patients. In addition, the information provided would provide useful support to the process of patient-physician shared decision making [18]. This approach would interrogate data to suggest next step options and weigh the risks and benefits of a treatment or test for a specific patient, the Holy Grail of personalized medicine.

## Discussion

### Required Data and Information

The most basic requirement for the DCDM system is the complete digital capture of patient information. We would maintain that de-identified clinical data constitutes a public good and should reside in a carefully managed public domain database, overseen by a cooperative coalition of vendors, provider institutions, and regulators. This is already the case for federally funded research data in the United States, and a movement is underway to share participant-level data from clinical trials [19-21]. Furthermore, the Patient Centered Outcomes Research Institute in the United States has already begun to develop the infrastructure that will aggregate large amounts of de-identified patient data from diverse sources for the purposes of observational research studies [22]. Any central database or federated query system must of course be governed by policies that account for the interests and preferences of the public regarding patient privacy, and the purposes for which the data are used [23]. The costs of database management would be built into purchase and maintenance agreements. Subsequent analyses would identify the clinical and financial impact of the entire data-based system with adjustments made as necessary.

In a DCDM system, a search engine would accept both structured and unstructured search terms to query the population database, much as current search engines query the database of the entire Internet. The unstructured terms could be used in a query via real time natural language processing or the next generation of “text to code” conversion applications, which convert free text to coded, structured search terms, while considering the context provided by free text, in order to ensure accuracy and clinical intention. The individual’s data would be rapidly compared to the population database to capture a set of useful records that match the content and context of the care encounter.

We envision every patient’s health data digitally catalogued according to demographics, diagnoses, treatments, and outcomes, all time stamped for sequential interpretation. We suspect the types of data included will evolve rapidly over time. For example, future data may be derived from cell phones or home monitors. This will be the basis of a data-based learning system of care where choices are made on the basis of substantial data, statistical support programs, and documented outcomes, rather than on individual experience and inconsistent use of applicable informational resources.

### Potential Obstacles

A significant caveat is that bias and/or residual confounding by indication may mar the analysis. The goal is to identify patient records in the database that are as similar as possible to the patient in terms of the variables that can confound the relationship between the intervention and the outcome as identified by clinician heuristics and complemented by computer algorithms, and then to compare the outcomes of those who receive the intervention versus those who did not. Residual confounding means that the outcome difference might not be due to the intervention, but rather due to something inherent to those patients who receive the treatment, or their condition. Realizing that the system is to be used by clinicians rather than data scientists, it must be designed so that such confounding and bias are minimized, with the confidence levels around the estimate of the treatment effects quantified and explained at the clinical user interface level.

The use of raw data from a variety of sources will present challenges. We acknowledge the inherent heterogeneity of people and disease. This presents an issue in terms of the levels of detail that require capture. The integration of data from multiple sources will require the use of standard terminologies and ontologies to allow for compatibility of the data from one source to another [14]. With the use of such standardization, these heterogeneities become inconveniences, not obstacles, to the vision. We foresee the implementation of progressively better EMRs, networks, and databases, all used by a generation of clinicians who have grown up with, are comfortable with, and expect to use and benefit from digital tools. It is important to anticipate potential risks, but this should be done in order to design and build the system so as to minimize them.

CDS tools must be engineered purposefully into workflow to avoid actually increasing user time and work requirements. An author of this paper (LAC) has previously reported on the use of local databases for the creation of CDS tools [24-26], which is one of the “grand challenges” in CDS [27]. Recent work adds the input of dynamic variables, which capture more information than traditional prediction models, including data on changes and variability of repeatedly measured values [28]. The readers are hereby directed to a recent review of the use of data mining in CDS [29]. DCDM would extend the capabilities of CDS by dynamically incorporating both individual and population data in real time [30,31]. In addition to querying and populating local databases, DCDM would also use the power of search engine technology to leverage population level data.

### Organization and Actuation

Combined clinical and engineering teams would need to work together to generate algorithms to determine the weight of each feature being matched against the outcome of interest, as well as the relative value of (and permissible missing values for) the interacting data elements in the match process. These algorithms should be modifiable in order to meet the continuously changing practice of medicine. Search engine algorithms are modifiable and these modifications can be engineered for specific purposes. Google has made a number of such strategic modifications to its algorithms over time [32]. It is likely that a prototype employing a smaller search target such as the Multiparameter Intelligent Monitoring in Intensive Care (MIMIC) Database [33] would be required to demonstrate the practicality and utility of the concept, as well as to create, develop, and initially refine the search engine algorithm. Indeed, the MIMIC Database has been previously employed to predict fluid responsiveness among hypotensive patients [34], as well as the hematocrit trend among patients with gastrointestinal bleeding [35], using the trajectory of physiologic variables over time.

The system would identify and suggest prioritized interventions and other courses of action that have been shown to be most valuable in terms of outcome and cost. The system’s features might include displays of quantitative and qualitative description of the match, hyperlinks that allow the user to drill further into the underlying data that is returned, and links to conventional practice guidelines and evidence-based modalities.

A clinical decision must be made at one point in time, but in most cases, decisions are ongoing and iterative. The system will incorporate the short-term response to the prior intervention each time that the system is accessed, but also capture long-term outcomes. For example, when a physician orders an intervention in response to acute kidney injury, the system would log the short-term response in serum creatinine, and also the long-term outcome of progression to or prevention of end-stage renal disease. The system could also be independently data mined to identify patterns that indicate whether the patient course is on track toward the desired outcome.

Large, diverse, international populations would improve the opportunity to achieve matches. When no match is possible, an alert could be provided noting the unusual features that preclude a match. The system would then provide appropriate suggestions for the user, such as a specialty referral, a data error of some kind, or even the possible detection of an entirely new condition. It would also serve as an epidemiological tool that recognizes emerging or spreading contagions [36,37], or other harmful exposures [38,39] more quickly and efficiently than is currently possible.

## Conclusion

DCDM has its roots in the need for medical care to be more fully based on data. The universal collection of data would also present the additional advantages of providing future opportunities to formulate randomized registry trials, as well as for other directed data mining purposes [40]. DCDM would begin to transform the exigent data entries that clinicians perform on a daily basis into a real tool for clinical care. Decisions would be made on the basis of experience over vast populations, rather than solely on individual knowledge and experience. We propose the creation of a system that supports clinician decision makers so that their decisions can be as logical, transparent, and unambiguous as possible. DCDM would more gainfully employ the power of networked computers, search engines, and data storage advances to leverage the copious, but underused data entered into EMRs.

## Abbreviations

CDS: clinical decision support
DCDM: dynamic clinical data mining
EMRs: electronic medical records
ICU: intensive care unit
MIMIC: Multiparameter Intelligent Monitoring in Intensive Care
SHARPn: Strategic Health Information Technology Advanced Research Project on secondary use
